# Effect of acute ketosis on lipid profile in prediabetes: findings from a cross-over randomized controlled trial

**DOI:** 10.1186/s12933-022-01571-z

**Published:** 2022-07-23

**Authors:** Yutong Liu, Sakina H. Bharmal, Wandia Kimita, Maxim S. Petrov

**Affiliations:** School of Medicine, University of Auckland, Auckland, New Zealand

**Keywords:** Prediabetes, Acute ketosis, Lipid profile, Remnant cholesterol, Triglycerides, Cardiovascular risk, Saturated fat

## Abstract

**Background:**

Ketone monoester β-hydroxybutyrate (KEβHB) ingestion has emerged as an effective method of inducing acute ketosis. Although evidence suggests that KEβHB can offer several therapeutic benefits, whether KEβHB affects lipid profile is still unknown.

**Aims:**

The primary aim was to study the effect of KEβHB on plasma lipid profile in individuals with prediabetes. The secondary aim was to investigate the role of saturated fat intake in that effect.

**Methods:**

This study was a randomized controlled trial with cross-over design. Following an overnight fast, 18 adults (six women and 12 men) with prediabetes (diagnosed based on the American Diabetes Association criteria) ingested a single dose of KEβHB drink or placebo drink. Blood samples were collected every 30 min, from baseline to 150 min. Outcome variables included changes in total cholesterol, high-density lipoprotein (HDL) cholesterol, low-density lipoprotein (LDL) cholesterol, remnant cholesterol, triglycerides, and the triglycerides to HDL cholesterol ratio. The area under the curve (AUC) over 150 min was calculated for each outcome following ingestion of the drinks. Habitual saturated fat intake was ascertained using the EPIC-Norfolk food frequency questionnaire.

**Results:**

Significant elevation of blood β-hydroxybutyrate from 0.2 mmol/L to 3.5 mmol/L (p < 0.001) was achieved within 30 min. Acute ketosis resulted in significantly lower AUCs for remnant cholesterol (p = 0.022) and triglycerides (p = 0.022). No statistically significant differences in the AUCs for total cholesterol, HDL cholesterol, LDL cholesterol, and the triglycerides to HDL cholesterol ratio were found. The changes in remnant cholesterol and triglycerides were statistically significant in individuals with high, but not low, habitual saturated fat intake.

**Conclusion:**

Acute ketosis had no untoward effect on plasma lipid profile. Moreover, it led to significantly reduced circulating levels of remnant cholesterol and triglycerides. This paves the way for investigating whether exogenous ketone supplementation reduces cardiovascular disease risk (via its actions on triglyceride-rich lipoproteins) in at-risk populations.

Trial registration: ClinicalTrials.gov, NCT03889210.

**Supplementary Information:**

The online version contains supplementary material available at 10.1186/s12933-022-01571-z.

## Introduction

Ketone bodies, in the form of β-hydroxybutyrate (βHB), acetoacetate, and acetone, are produced in the liver from fatty acids (released from adipose tissue) as an alternative energy source to glucose in response to fasting or carbohydrate restriction – a process called ‘endogenous ketosis’ [1]. Mild nutritional ketosis is beneficial in terms of aiding weight loss [2], reducing seizure events in recalcitrant epilepsy [3], and improving blood glucose control [4]. Until recently, nutritional ketosis has only been achieved by strict compliance with high-fat low-carbohydrate diets (i.e., ketogenic diets). However, these are often associated with metabolic acidosis and gastrointestinal symptoms [5, 6]. Further, the long-term adherence to ketogenic diets is typically limited due to their poor palatability and restrictive nature [7]. Exogenous ketones, in the form of either ketone monoester or ketone salts, have recently been designed as a possible alternative to ketogenic diets. In particular, ketone monoester drink containing D-β-hydroxybutyrate-R-1.3-butanediol (KEβHB) was shown to elevate the circulating levels of βHB to as high as 5 mmol/L within 30 min of ingestion in healthy individuals [8, 9] as well as those with metabolic disorders [10, 11].

Given the high ratio of calories as dietary fat (up to 90% daily energy), one common concern with the use of ketogenic diets is its propensity to causing dyslipidaemia – a major cardiovascular risk factor [12]. For example, a meta-analysis of 13 randomized controlled trials (RCTs) showed that the use of ketogenic diets was significantly associated with an increase in low-density lipoprotein (LDL) cholesterol [2]. Further, unfavourable changes in lipid profile were reported in a study of ketogenic diet high in saturated fat [13] – a well-acknowledged risk factor for cardiovascular disease [14]. However, when it comes to exogenous ketones, to date there has been no RCT examining the effect of acute ketosis on the standard lipid panel (i.e., triglycerides, total, high-density lipoprotein (HDL), and LDL cholesterol). A 2019 observational study found no significant differences in the standard lipid panel before and after 28 days of exogenous ketone supplementation, though it is worth noting that the study included both sedentary adults and athletes (some of whom also followed a ketogenic diet) [15]. One component of the standard lipid panel, LDL cholesterol, is typically calculated (rather than measured directly). In this regard, the 2020 Sampson formula has been proven to be more accurate than other methods of calculating LDL cholesterol [16] but very few RCTs have used it thus far. Further, several parameters derived from the standard lipid panel have been found useful in cardiovascular medicine, yet have never been studied in RCTs of exogenous ketones. These include remnant cholesterol (i.e., the cholesterol content of triglyceride-rich lipoproteins) – a causal risk factor for ischemic heart disease [17] and coronary artery disease [18], and the triglyceride to HDL cholesterol ratio – an independent predictor of cardiovascular disease [19, 20]. As the presence of abnormal glucose metabolism heightens the risk of cardiovascular disease [21], there is an unmet need to elucidate the effect of acute ketosis on lipid profile in this category of people.

The present study aimed to investigate the effect of KEβHB on plasma lipid profile in individuals with prediabetes. The role of habitual saturated fat intake was also studied.

## Methods

### Trial design

The study was part of the CETUS (Cross-over randomisEd Trial of β-hydroxybUtyrate in prediabeteS) project and represented a pre-specified analysis of secondary outcomes (specifically, changes in plasma lipid profile). The CETUS project was a cross-over, placebo-controlled, randomized trial investigating the effect of KEβHB. The project was approved by the New Zealand Health and Disability Ethics Committee (18/NTB/161) and was prospectively registered at www.ClinicalTrials.gov (NCT03889210). The findings related to the primary outcome – change in plasma glucose levels – were reported elsewhere [10].

### Eligibility criteria

Eligible participants were individuals of 18 years old and above, who had a diagnosis of prediabetes according to the guidelines published by the American Diabetes Association: fasting plasma glucose between 100 and 125 mg/dL (5.6–6.9 mmol/L) and/or glycated haemoglobin between 5.7% and 6.4% (39–47 mmol/mol) [22]. The exclusion criteria were malignancy, history of bariatric or another gastrointestinal surgery, following a ketogenic diet or consuming a nutritional ketone supplement, participation in competitive sports or intensive endurance training, and women who were pregnant or lactating. None of the study participants received antidiabetic or lipid-lowering medications or had a known cardiovascular disease.

### Intervention

Participants were requested to refrain from alcohol and exercise for at least 24 h prior to each clinic visit. They were asked to consume a similar meal the night before each visit. The study took place at the COSMOS clinic of the University of Auckland (www.cosmos.auckland.ac.nz), where participants attended each visit following an overnight fast of at least 8 h [23]. Eligible participants were randomly assigned to ingesting a dose containing 4.4 mmol/kg of D-β- hydroxybutyrate (equivalent to 1.05 ml/kg and 1.9 kcal/kg of individual lean body weight) or a placebo drink comprising of water, flavoured stevia, malic acid, and arrowroot (which contained no calories). The drinks were isovolumic (100 mL) and had similar taste, colour, viscosity. Participants were asked to remain sedentary throughout their clinic visit. Venous blood samples were collected at baseline (fasted) and at intervals of 30 min for 150 min following the ingestion of KEβHB/placebo drink. Participants were also asked to fill out a food frequency questionnaire and a standardised questionnaire assessing their consumption of alcohol and tobacco smoking [24]. The above protocol was repeated for the second clinic visit, except participants ingested the alternative drink.

### Outcomes

Fresh, never frozen venous blood samples at baseline and five time points after the ingestion of drinks (30, 60, 90, 120, and 150 min) were collected into 4.5 ml plasma separator tubes and immediately sent to LabPlus for the measurement of total cholesterol, HDL cholesterol, and triglycerides. LabPlus is an accredited tertiary medical laboratory located in Auckland City Hospital (New Zealand). Although LabPlus performed calculation of LDL cholesterol using the Friedewald formula, LDL cholesterol levels were re-calculated for the purpose of the present study by the research team using the Sampson formula [16]. In addition, remnant cholesterol was calculated as total cholesterol minus HDL cholesterol minus LDL cholesterol (using the Sampson formula) [25]. The ratio of triglycerides to HDL cholesterol was calculated as the levels of triglycerides in mmol/L divided by HDL cholesterol in mmol/L [26]. A handheld ketone meter and FreeStyle Optium β-ketone test strips (Abbott Laboratories, Illinois, USA) were used to measure βHB in whole blood.

#### Assessment of saturated fat intake

Participants were asked to complete the EPIC-Norfolk food frequency questionnaire – a robust internationally validated method of assessing habitual food intake by collecting data on the consumption of 130 food items [27]. They were instructed to indicate their frequency of consumption from nine frequency categories as a part of their habitual diet, ranging from ‘never or less than once/month’ to ‘more than six per day’. Data were processed using the CAFE (Compositional Analyses from Frequency Estimates) software.

#### Assessment of covariates

Cardiovascular disease risk was estimated using the atherosclerotic cardiovascular disease risk algorithm endorsed by the American College of Cardiology and the American Heart Association [28]. The algorithm predicted participants’ 10-year risk of heart disease or stroke from the parameters such as age, sex, race, blood pressure, total cholesterol, HDL cholesterol, and smoking status. The algorithm was incorporated into the U.S. Preventive Service Task Force guidelines for the prevention of cardiovascular disease [29]. Lipoprotein lipase was measured in baseline samples using enzyme-linked immunosorbent kit (Cloud-Clone Corp., USA).

### Randomization

The randomization sequence was created using an online generator of random numbers (www.sealedenvelope.com) with the allocation ratio of 1:1. Participants were randomly assigned to one of the two treatment groups in cross-over fashion. Neither the study participants nor the researchers running the trial were aware of the randomization sequence.

### Statistical methods

Statistical analyses were conducted using Prism software version 9 (GraphPad Software package), SPSS software version 28.0 (IBM Corporation), and SAS software version 9.4 (SAS Institute, Cary, NC, USA). All variables were checked for normality using the Shapiro–Wilk test prior to analysis. Baseline characteristics were described as median and interquartile range. Less than 1% of lipid profile and smoking variables had been missing and were imputed using multiple regression models with the use of SPSS software. All participants were first included in the overall analysis and then stratified into the ‘high’ and ‘low’ fat intake subgroups according to the median value of their habitual saturated fat intake. Three steps were involved. First, all variables were log-transformed prior to analyses. The trapezoid method was used to calculate the area under the curve (AUC) for each outcome from baseline (0 min) to 150 min. Comparisons of AUCs between the drinks (KEβHB versus placebo) were conducted by paired-sample t tests, and Cohen’s d was used to determine effect size. Second, repeated measures two-way analysis of variance with the Geisser-Greenhouse correction was used to assess the changes in outcomes over 150 min (time effect) following the KEβHB drink versus the placebo drink (treatment effect), along with the interaction between time and treatment (interaction effect). Third, paired-sample t test was used to compare the outcomes at individual time points. p values < 0.05 were deemed to be statistically significant in all analyses.

## Results

### Characteristics of study participants

**Fig. 1:**
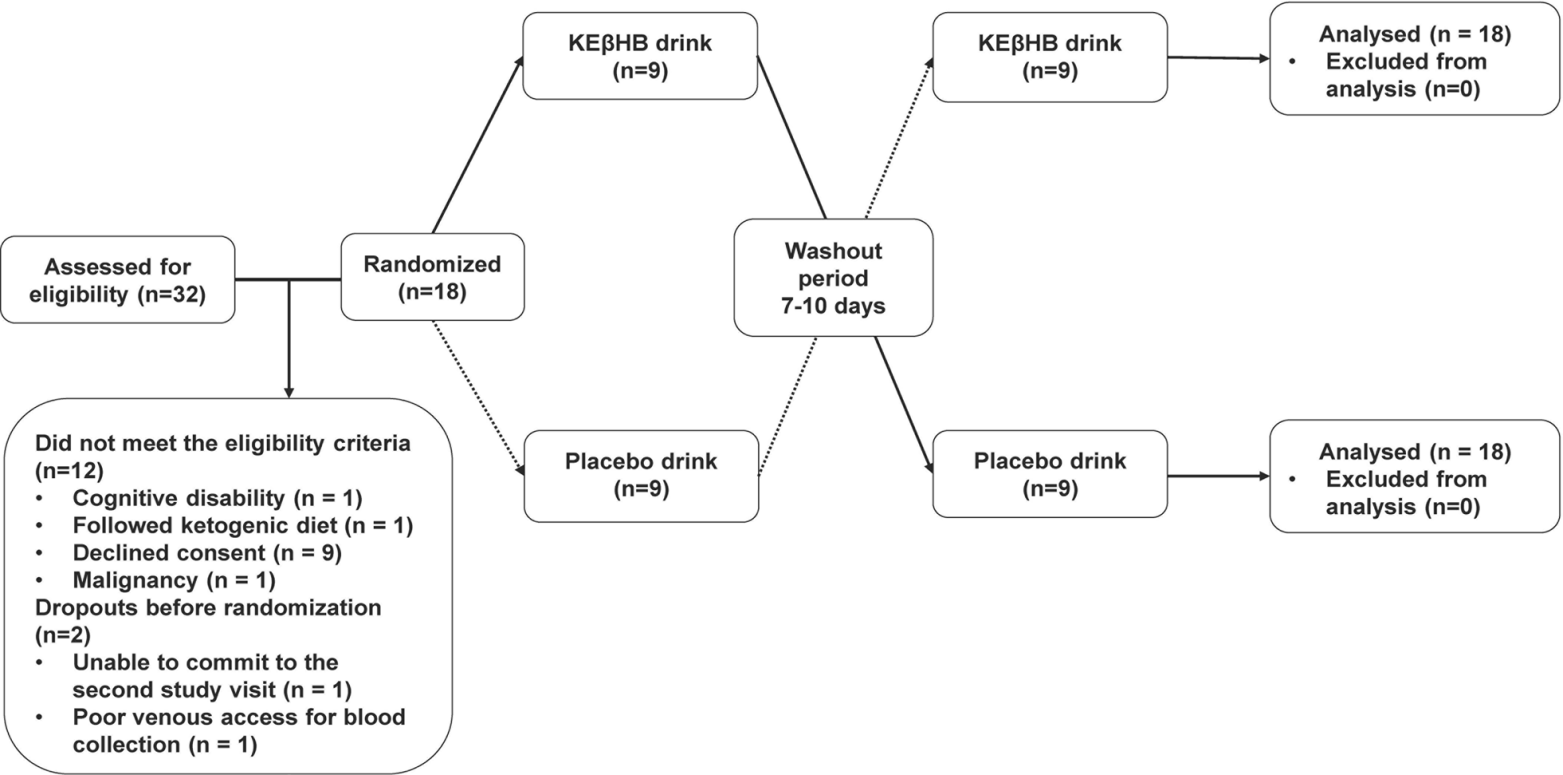
Consolidated Standards of Reporting Trials (CONSORT) diagram illustrating the flow of participants in the CETUS trial

Eighteen participants met the eligibility criteria and completed the trial (Table 1). No participant was excluded after randomization (Fig. 1). None of the study participants had deficiency of lipoprotein lipase. The median habitual saturated fat intake of the study participants was 27.38 g/day, ranging from 6.56 to 50.72 g/day. The median predicted cardiovascular disease risk of the study participants was 5.5%, ranging from 0 to 50%.

**Table 1:** Baseline characteristics

Characteristic	n = 18^1^
Age (years)	58.00 (45.50, 67.50)
Waist to hip ratio	0.92 (0.87, 0.98)
Systolic blood pressure (mmHg)	127.50 (113.80, 147.30)
Diastolic blood pressure (mmHg)	84.0 (76.50, 91.50)
Habitual intake of saturated fat (g/day)	27.38 (17.44, 38.85)
Glucose (mmol/L)	5.45 (5.15, 5.93)
Total cholesterol (mg/dL)	195.30 (163.40, 216.60)
HDL cholesterol (mg/dL)	46.40 (41.57, 52.34)
LDL cholesterol (mg/dL)	121.70 (88.32, 139.80)
Remnant cholesterol (mg/dL)	29.91 (19.50, 35.62)
Triglycerides (mg/dL)	168.30 (110.70, 199.30)
Triglycerides to HDL cholesterol ratio	1.59 (0.94, 2.19)
Lipoprotein lipase (ng/mL) Cardiovascular disease risk (%)	224.60 (89.05, 528.60) 5.50 (1.75, 11.30)

*HDL* high-density lipoprotein, *LDL* low-density lipoprotein

^1^Data are presented as median (interquartile range)

### Effect of the KEβHB versus placebo drinks on βHB

Following the KEβHB drink, the mean ± standard deviation levels of βHB were significantly elevated, in comparison with baseline (0.18 ± 0.07 mmol/L), at 30 min (3.47 ± 0.92 mmol/L, p < 0.001), 60 min (2.88 ± 0.46 mmol/L, p < 0.001), 90 min (2.17 ± 0.49 mmol/L, p < 0.001), 120 min (1.24 ± 0.41 mmol/L, p < 0.001), and 150 min (0.77 ± 0.33 mmol/L, p < 0.001). Following the placebo drink, no significant difference in the levels of βHB was observed at any time point in comparison with baseline (0.17 ± 0.07 mmol/L).

### Effect of the KEβHB versus placebo drinks on lipid profile

#### Total cholesterol

**Fig. 2:**
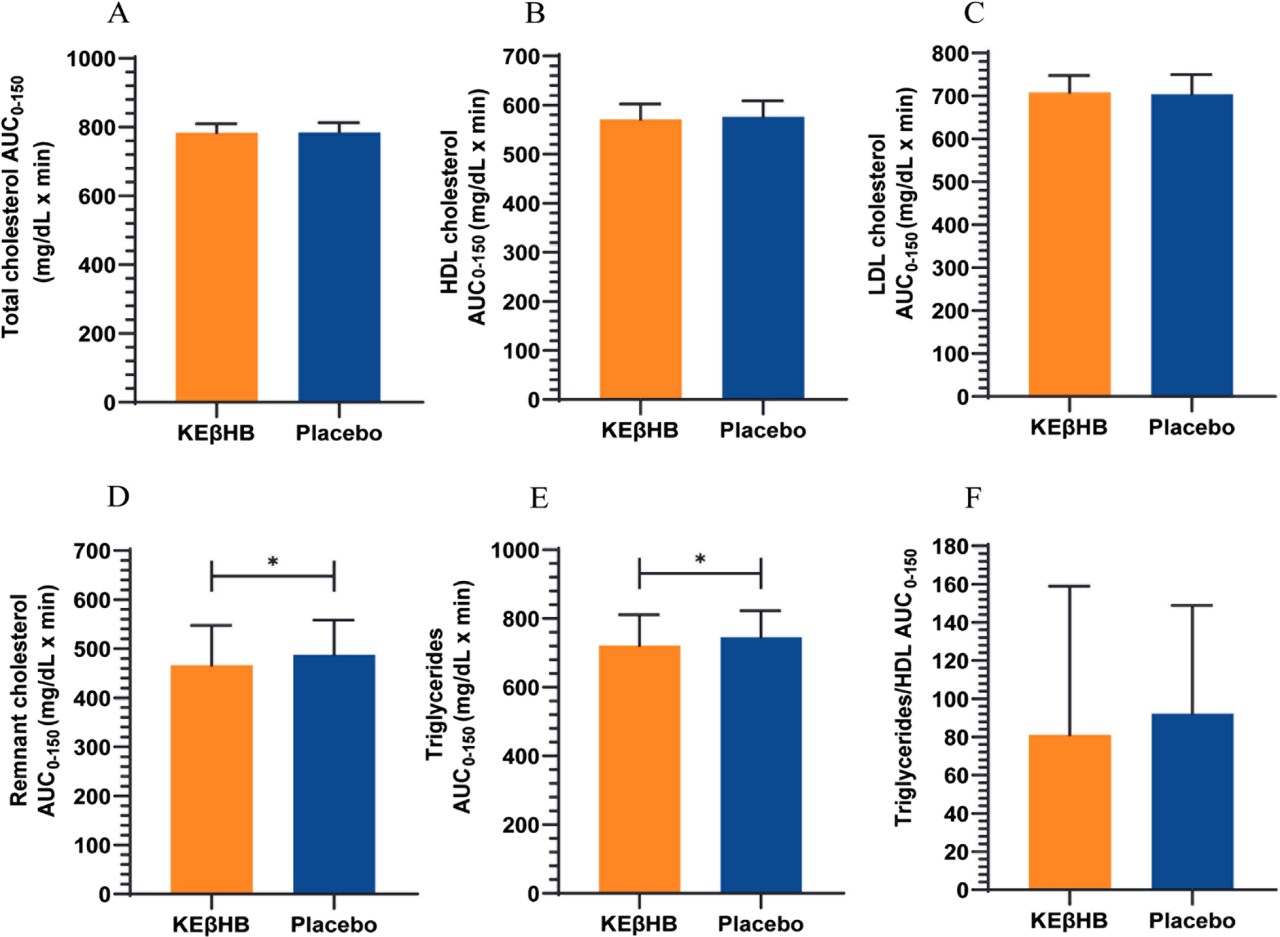
Effect of the KEβHB versus placebo drinks on **A** total cholesterol, **B** HDL cholesterol, **C** LDL cholesterol, **D** remnant cholesterol, **E** triglycerides, and **F** the triglycerides to HDL cholesterol ratio. Area under the curve (AUC_0-150_) data are presented as mean ± standard deviation. *p < 0.05 for the difference between the KEβHB and placebo drinks. *AUC* area under the curve; *HDL* high-density lipoprotein, *KEβHB* ketone monoester (β-hydroxybutyrate), *LDL* low-density lipoprotein

The AUCs_0-150_ for total cholesterol were 783.6 ± 25.61 mg/dL × min after the KEβHB drink versus 784.9 ± 27.78 mg/dL × min after the placebo drink (p = 0.682; d = 0.05) (Fig. 2). There were no significant interactions of time × intervention (p = 0.948) or main treatment effect (p = 0.992). However, there was a statistically significant main effect of time for total cholesterol (p = 0.045).

#### HDL cholesterol

The AUCs_0-150_ for HDL cholesterol were 570.9 ± 31.71 mg/dL × min after the KEβHB drink versus 575.8 ± 32.88 mg/dL × min after the placebo drink (p = 0.119; d = 0.15) (Fig. 2). There were no significant interactions of time × intervention (p = 0.432) or main treatment effect (p = 0.656) for HDL cholesterol. However, there was a statistically significant main effect of time for HDL cholesterol (p = 0.033).

#### LDL cholesterol

The AUCs_0-150_ for LDL cholesterol were 708.2 ± 39.64 mg/dL × min after the KEβHB drink versus 704.3 ± 45.43 mg/dL × min after the placebo drink (p = 0.407; d = 0.09) (Fig. 2). There were no significant interactions of time × intervention (p = 0.915), main treatment effect (p = 0.788), or main time effect (p = 0.150) for LDL cholesterol.

#### Remnant cholesterol

The AUCs_0-150_ for remnant cholesterol were statistically significantly lower after the KEβHB drink (466.5 ± 80.72 mg/dL × min) than after the placebo drink (487.7 ± 70.99 mg/dL × min) (p = 0.022, d = 0.28) (Fig. 2). There were no significant interactions of time × intervention (p = 0.095), main treatment effect (p = 0.419), or main time effect (p = 0.051) for remnant cholesterol.

#### Triglycerides

The AUCs_0-150_ for triglycerides were statistically significantly lower after the KEβHB drink (721.6 ± 89.63 mg/dL × min) than after the placebo drink (745.4 ± 77.77 mg/dL × min) (p = 0.022; d = 0.28) (Fig. 2). There was a statistically significant main effect of time for triglycerides (p = 0.030), but neither interactions of time × intervention (p = 0.092) nor main treatment effect (p = 0.411) was statistically significant.

#### Triglycerides to HDL cholesterol ratio

The AUCs_0-150_ for the triglycerides to HDL cholesterol ratio were 81.0 ± 77.82 mg/dL × min after the KEβHB drink versus 92.1 ± 56.99 mg/dL × min after the placebo drink (p = 0.217; d = 0.16) (Fig. 2). There were no significant interactions of time × intervention (p = 0.215), main treatment effect (p = 0.603), or main time effect (p = 0.124) for the triglycerides to HDL cholesterol ratio.

### Role of habitual saturated fat intake

#### High saturated fat intake

**Fig. 3:**
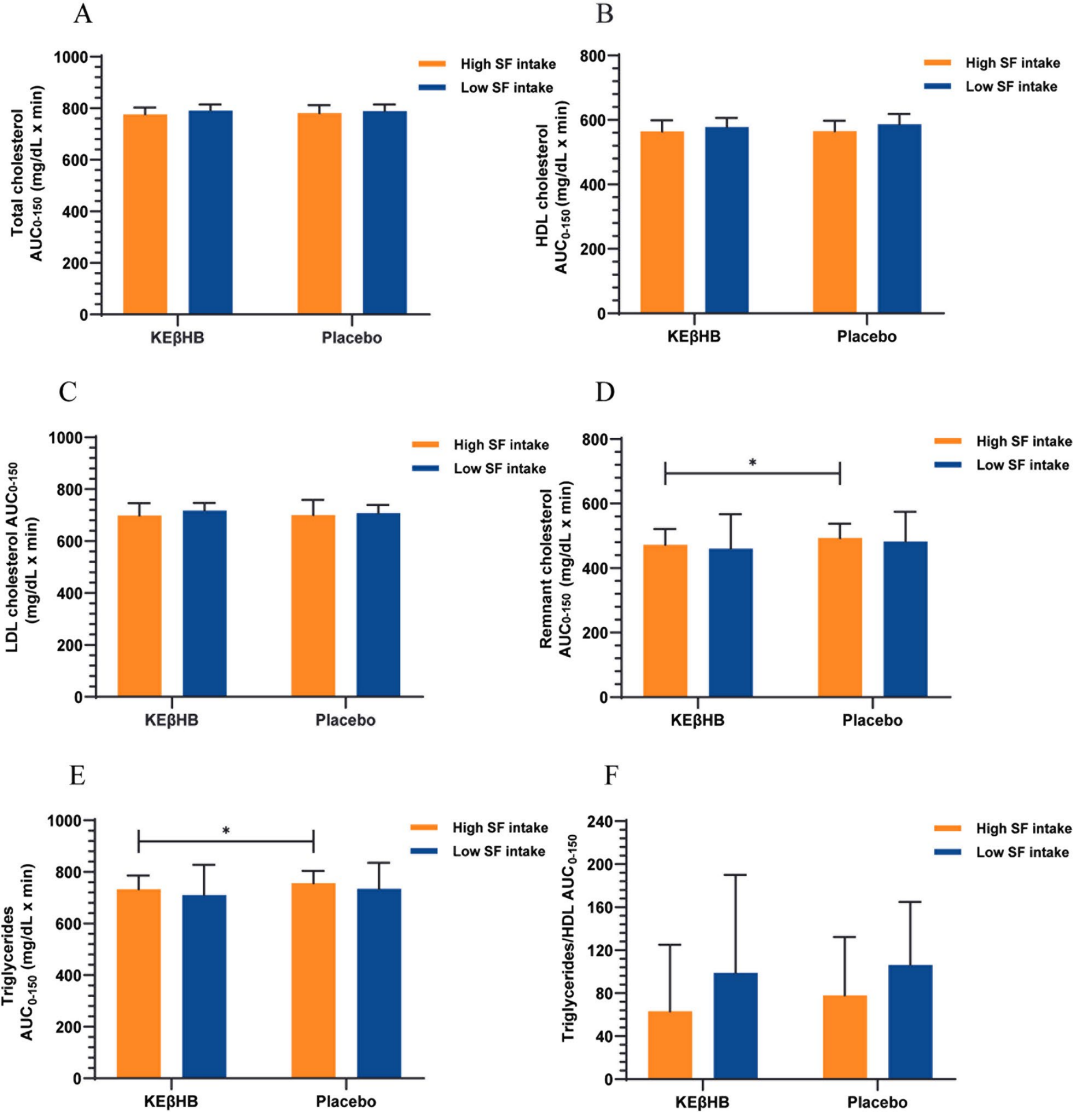
Effect of the KEβHB versus placebo drinks on lipid profile according to intake of saturated fat. Participants were stratified based on the median value of habitual dietary saturated fat intake. Area under the curve (AUC_0-150_) data are presented as mean ± standard deviation. *p < 0.05 for the difference between the KEβHB and placebo drinks. *HDL* high-density lipoprotein, *KEβHB* ketone monoester (β-hydroxybutyrate), *LDL* low-density lipoprotein, *SF* saturated fat

The AUCs_0-150_ were significantly lower after the KEβHB versus placebo drinks for remnant cholesterol (472.6 ± 48.73 mg/dL × min versus 493.2 ± 44.43 mg/dL × min; p = 0.041) and triglycerides (732.9 ± 53.29 mg/dL × min versus 756.1 ± 48.69 mg/dL × min; p = 0.041) in individuals with high saturated fat intake (Additional file 1: Fig. S1). No statistically significant difference was found when comparing the AUCs_0-150_ after the KEβHB versus placebo drinks for total cholesterol (p = 0.364), HDL cholesterol (p = 0.787), LDL cholesterol (p = 0.804), and for the triglycerides to HDL cholesterol ratio (p = 0.172) in individuals with high saturated fat intake (Fig. 3).

#### Low saturated fat intake

No statistically significant difference was found when comparing the AUCs_0-150_ after the KEβHB versus placebo drinks for total cholesterol (p = 0.497), HDL cholesterol (p = 0.074), LDL cholesterol (p = 0.127), remnant cholesterol (p = 0.190), triglycerides (p = 0.190), and the triglycerides to HDL cholesterol ratio (p = 0.629) in individuals with low saturated fat intake (Table 2).

**Table 2:** Effects of the KEβHB versus placebo drinks on lipid profile stratified by habitual intake of saturated fat

Outcome^1^	Group/significance^2^	Saturated fat intake	
		High	Low
Total cholesterol (mg/dL × min)	KEβHB (n = 18)	776.0 ± 26.97	791.2 ± 23.18
	Placebo (n = 18)	780.9 ± 30.94	788.8 ± 25.44
	Δ (KEβHB-placebo)	−4.86 (−16.48, 6.77)	2.32 (−5.21, 9.85)
	d	0.17	0.10
	p	0.36	0.50
HDL cholesterol (mg/dL × min)	KEβHB (n = 18)	564.0 ± 34.87	577.7 ± 28.56
	Placebo (n = 18)	565.1 ± 32.22	586.4 ± 31.68
	Δ (KEβHB-placebo)	−1.14 (−10.59, 8.30)	−8.72 (−18.50, 1.06)
	d	0.03	0.29
	p	0.79	0.07
LDL cholesterol (mg/dL × min)	KEβHB (n = 18)	698.8 ± 47.74	717.6 ± 29.34
	Placebo (n = 18)	700.7 ± 57.96	708.0 ± 31.56
	Δ (KEβHB-placebo)	−1.81 (−18.11, 14.48)	9.64 (−3.41, 22.70)
	d	0.04	0.32
	p	0.80	0.13
Remnant cholesterol (mg/dL × min)	KEβHB (n = 18)	472.6 ± 48.73	460.5 ± 106.7
	Placebo (n = 18)	493.2 ± 44.43	482.1 ± 93.09
	Δ (KEβHB-placebo)	−20.68 (−40.30, −1.06)	−21.57 (−56.25, 13.11)
	d	0.44	0.22
	p	0.04*	0.19
Triglycerides (mg/dL × min)	KEβHB (n = 18)	732.9 ± 53.29	710.2 ± 118.1
	Placebo (n = 18)	756.1 ± 48.69	734.6 ± 101.1
	Δ (KEβHB-placebo)	−23.19 (−45.22, −1.16)	−24.39 (−63.61, 14.83)
	d	0.45	0.22
	p	0.04*	0.19
Triglycerides to HDL cholesterol ratio	KEβHB (n = 18)	63.1 ± 61.92	98.9 ± 91.19
	Placebo (n = 18)	77.9 ± 54.37	106.3 ± 58.48
	Δ (KEβHB-placebo)	−14.81 (−37.58, 7.97)	−7.41 (−41.47, 26.64)
	d	0.25	0.10
	p	0.17	0.6

*HDL* high-density lipoprotein, *KEβHB* ketone monoester (β-hydroxybutyrate), *LDL* low-density lipoprotein

^1^Outcomes were area under the curves of log-transformed variables calculated from 0 to 150 min

^2^Mean differences (95% confidence intervals), d values, and p values were obtained from paired t test. Values are presented as mean ± standard deviation

## Discussion

This was the first RCT investigating the effect of acute ketosis on the standard lipid panel in humans. Although effects of acute ketosis on a wide array of blood biomarkers were investigated in numerous studies, only two RCTs (one of which was not placebo-controlled) studied the effect of acute ketosis on levels of triglycerides alone (but not the standard lipid panel) [11, 30]. In the present CETUS trial, participants were put into acute ketosis within 30 min of ingesting the KEβHB drink, as their βHB levels in blood became significantly elevated from 0.18 mmol/L to 3.47 mmol/L. Participants also remained in the state of ketosis for the entire duration of the trial. One notable finding of the CETUS trial was that the KEβHB drink did not have a negative impact on plasma lipid profile. The other notable finding was that the KEβHB drink significantly lowered the levels of remnant cholesterol and triglycerides in comparison with the placebo drink. Further, the KEβHB drink exerted its effect in participants with high (but not low) habitual saturated fat intake.

Nutritional ketosis achieved by means of ketogenic diets was shown to have an inconsistent effect on the standard lipid panel. Some studies demonstrated either an increase [31] or no significant change in total [32, 33] and LDL cholesterol [34] levels following a ketogenic diet. By contrast, other studies showed a reduction in total [35, 36] and LDL cholesterol [37], as well as an increase in HDL cholesterol levels in healthy [38] and obese [39, 40] individuals. It is worth noting that there were important differences in terms of macronutrient composition of the studied diets though. For example, while increase in LDL cholesterol was notable in the studies where ketogenic diets were high in saturated fats by design [35], increase in HDL cholesterol was observed in the studies of ketogenic diets rich in unsaturated fat [41]. The only previous study that investigated the effect of exogenous ketosis on the standard lipid panel was observational. A total of 24 healthy adults (both athletes and sedentary adults) received a total of 2.1 L of KEβHB over 28 days and no negative effect on lipid profile was observed [15]. The CETUS trial provides a complementary evidence from acute study on the consistent absence of untoward effect of KEβHB on levels of total, HDL, and LDL cholesterol. Moreover, unlike any of the above-mentioned studies, LDL was calculated using the most accurate 2020 Sampson formula in the present study. Taken together, the two studies establish the safety of exogenous ketone supplementation in regard to lipid profile.

The CETUS trial also investigated, for the first time, the effect of acute exogenous ketosis on remnant cholesterol. We found a significantly reduced remnant cholesterol (as well as triglycerides) following the KEβHB drink. Remnant cholesterol consists of very low-density LDL, intermediate-density lipoprotein, and chylomicron remnants. [42] Of note, the contribution of the lattermost to remnant cholesterol levels in the CETUS trial was negligible as all participants were studied after an overnight fast. Similar to elevated triglycerides [43], remnant cholesterol has been recently given prominence as an independent causal risk factor for cardiovascular disease [44]. Our findings are in line with the results of a small number of earlier studies of ketogenic diets. Those studies reported on significantly reduced levels of remnant cholesterol in individuals with diabetes [45] and significantly reduced levels of triglycerides in obese individuals [46] on ketogenic diets. Taken together, the above studies indicate that nutritional ketosis (via either ketogenic diet or exogenous ketone supplementation) results in reduced circulating levels of triglyceride-rich lipoproteins, which likely has a beneficial effect on the cardiovascular system. However, not all individuals in the state of nutritional ketosis are positioned equally well to have their cardiovascular disease risk reduced. This is because, although the reduction in levels of remnant cholesterol was statistically significant in the CETUS trial, the effect size of 0.28 was rather small. Similarly, while acute ketosis significantly lowered the levels of triglycerides, most of our study participants had their baseline triglycerides levels below 200 mg/dL (2.2 mmol/L), which are not thought to be associated with increased cardiovascular disease risk. However, it is worth noting that, when we replicated the analyses in individuals with high habitual intake of saturated fat (a well-established dose-dependent risk factor for cardiovascular disease [47]), the effect size for remnant cholesterol increased markedly to 0.44. It is therefore conceivable that a clinically relevant reduction of cardiovascular disease risk resulting from nutritional ketosis is more pronounced in people with disorders other than prediabetes (e.g., atherogenic dyslipidaemia, remnant removal disease).

Emerging evidence suggests that remnant cholesterol contributes to atherosclerosis through a mechanism other than LDL cholesterol [44]. One population-based study found a strong association between elevated remnant cholesterol and increased C-reactive protein (a well-known marker of low-grade inflammation), as well as the risk of ischaemic heart disease [48]. At the same time, there was no significant association between LDL cholesterol and C-reactive protein. As opposed to LDL cholesterol, remnant cholesterol is thought not to require oxidative modifications before being taken up by macrophages [49], making it equivalently or even more atherogenic than LDL cholesterol [51]. Fish oil supplements – mainly omega-3 fatty acids (eicosapentaenoic acid and docosahexaenoic acid) – have been shown to decrease cardiovascular disease risk and mortality [50], as well as circulating levels of remnant cholesterol (and triglycerides) with [52] and without [53] concurrent lipid-lowering therapy (such as statins). Taking into account the beneficial effect of add-on triglyceride-rich lipoprotein lowering n-3 fatty acids therapy found in the 2019 REDUCE-IT trial in individuals already receiving statins [54], the findings of the CETUS trial suggest that exogenous ketone supplementation could be considered as another therapy targeted at triglyceride-rich lipoproteins in high-risk populations.

The present study has several strengths. First, we used the NIH-endorsed 2020 Sampson formula rather than the now outdated ‘one size fits all’ Friedewald formula for calculating LDL cholesterol (and remnant cholesterol). The former surpasses the Friedewald formula not only in terms of higher accuracy when plasma triglycerides are low, but also when its levels are up to 800 mg/dL (9 mmol/L) [16]. Second, to minimise possible inaccuracies resulting from long-term blood sample storage, fresh never frozen blood samples were used for the analysis of lipid profile immediately following blood collection [55]. Further, the standard lipid panel was analysed in a tertiary referral laboratory (as opposed to the use of a point-of-care device or immunoassays conducted by researchers). Last, we investigated the effect of KEβHB without introducing other nutrient stimulants (e.g., fat, carbohydrate). This was an issue in an earlier study where participants consumed a drink with 30% energy as exogenous ketone (and 32% energy as carbohydrate, 19% as protein, and 19% as fat), making it difficult to rule out the possible confounding effects of macronutrients [56].

Limitations of the present study are to be acknowledged. First, while we demonstrated statistically significant differences in terms of remnant cholesterol and triglycerides, the CETUS trial was not powered to investigate differences in lipid profile. Therefore, a type II error in regard to the other studied components of lipid profile cannot be ruled out. Second, although we presented data on all parameters of the standard lipid panel (along with two derivatives), we did not investigate the size and number of LDL particles or apolipoproteins – other established predictors of cardiovascular events [45, 57, 58]. However, we did study the triglycerides to HDL cholesterol ratio, which is known to correlate inversely with circulating levels of small dense LDL [20]. Nevertheless, purposely-designed studies are warranted to investigate the effect of acute ketosis on size and number of LDL particles as well levels of apolipoproteins. Third, remnant cholesterol was calculated from the standard lipid panel rather than measured directly. The former approach may underestimate or overestimate the actual levels of triglyceride-rich lipoproteins depending on levels of triglycerides [59]. Future studies may consider using direct measurement of remnant cholesterol. Fourth, only blood lipids (cholesterol and triglycerides) were investigated in the present study. The effect of acute ketosis on free fatty acids – another risk factor for cardiovascular disease – could be of interest. However, investigating these in the present study would have been redundant as several previous RCTs found free fatty acids to be significantly reduced following acute ketosis (whereas no previous RCT used the standard lipid panel as an outcome) [11, 60–62]. Last, lipid profile is altered in individuals with remnant removal disease or lipoprotein lipase deficiency (which were not our exclusion criteria). However, we measured lipoprotein lipase in all study participants, and none had low circulating levels of this enzyme. Further, the cross-over study design mitigates the possible effect of a genetic dyslipidaemia.

## Conclusion

The CETUS trial demonstrated the first evidence that exogenous ketosis significantly reduces the levels of remnant cholesterol and triglycerides in plasma. This provides a proof of concept that triglyceride-rich lipoproteins can be effectively targeted by nutritional ketosis with a view to reducing cardiovascular disease risk.

## Supplementary Information

**Additional file 1: Figure S1.** Effect of the KEβHB versus placebo drinks on lipid profile at individual time points.

## Data Availability

Available upon reasonable request.
