# Supplementary material for: Effect of acute ketosis on lipid profile in prediabetes: findings from a cross-over randomized controlled trial
Source: Cardiovasc Diabetol. 2022 Jul 23;21:138. doi: 10.1186/s12933-022-01571-z (PMC9308353; doi:10.1186/s12933-022-01571-z)
Supplement: Supplementary file 1 — Additional file 1: Figure S1. Effect of the KEβHB versus placebo drinks on lipid profile at individual time points. [file 12933_2022_1571_MOESM1_ESM.docx]

A B

C D

E F

***Supplementary figure 1.*** Effect of the KEβHB versus placebo drinks on lipid profile at individual time points

*Footnotes: Participants were stratified according to the median value of habitual dietary saturated fat intake. Data are presented as median and interquartile range at 0 (fasted), 30, 60, 90, 120, and 150 minutes.*

*Abbreviations: HDL, high-density lipoprotein; KEβHB, ketone monoester (β-hydroxybutyrate); LDL, low-density lipoprotein; SF, saturated fat.*
